# Effect of Personalized Outreach on Medicaid to Marketplace Coverage Transitions: A Randomized Clinical Trial

**DOI:** 10.1001/jamahealthforum.2022.3616

**Published:** 2022-10-14

**Authors:** Katie Ravel, Jahan Ahrary, Karen Avakian, Andrew Feher, Isaac Menashe

**Affiliations:** 1 Covered California, Sacramento

## Abstract

This randomized clinical trial examines the effect of email reminders, personalized telephone outreach, and a combination of both on Affordable Care Act enrollment among households who recently lost Medicaid and became eligible for subsidized marketplace coverage in 2017 in California.

## Introduction

Amid projections that upwards of 15 million people could lose Medicaid eligibility once the COVID-19 public health emergency expires, there is a need to identify effective outreach strategies that states can use to facilitate Medicaid to Affordable Care Act (ACA) coverage transitions.^1^ In contrast to prior randomized clinical trials that have tested singular outreach approaches (eg, letter or phone call), we examine the effect of email reminders, personalized telephone outreach, and the combination of the 2 forms of outreach on ACA enrollment among households who recently lost Medicaid and became eligible for subsidized marketplace coverage during a special enrollment period in 2017 in California’s ACA marketplace.^2,3,4^

## Methods

This randomized clinical trial followed the Consolidated Standards of Reporting Trials (CONSORT) reporting guideline and was approved by the State of California Health and Human Services institutional review board, which did not require informed consent (Supplement 1). A total of 3674 households were screened for eligibility, and 1501 were excluded due to subsidy ineligibility, yielding a sample of 2173 households no longer eligible for Medicaid but instead determined eligible for subsidized coverage through Covered California, the state’s ACA marketplace (eFigure in Supplement 2). At the end of August 2017, we randomly assigned households to 1 of 4 groups based on the last digit of their household identifier: a control group assigned to receive no outreach beyond the required eligibility determination notice provided to households based on their communication preference; an email-only group assigned to receive the required notice plus email reminders about signing up for marketplace coverage; a phone-only group assigned to receive the required notice plus a phone call offering enrollment assistance from a service center representative; and a phone-plus-email group assigned to receive the required notice, email reminders, and a phone call offering enrollment assistance (Table).

**Table.** Baseline Demographic Characteristics for Each Group

Characteristic	%			
	Control	Email	Phone	Phone and email
No.	440	873	432	428
Mean (SD) age, y	32.8 (14.3)	33.2 (15.1)	31.9 (15.6)	33.4 (15.8)
Federal poverty level, mean (SD) %	182.7 (62.0)	184.4 (63.2)	182.2 (64.3)	183.4 (64.4)
Race and ethnicity, No. (%)^a^				
Asian	38 (8.6)	77 (8.8)	50 (11.6)	48 (11.2)
Black	27 (6.1)	44 (5.0)	17 (4.0)	23 (5.4)
Latino	233 (53.0)	451 (51.7)	214 (49.5)	206 (48.1)
Non-Hispanic White	74 (16.8)	172 (19.7)	89 (20.6)	90 (21.0)
Other/unknown^b^	68 (15.5)	129 (14.8)	62 (14.4)	61 (14.3)
Sex, No. (%)				
Female	255 (58.0)	531 (60.8)	251 (58.1)	243 (56.8)
Male	185 (42.0)	324 (39.2)	181 (41.9)	185 (43.2)
Language preference, No. (%)				
English	287 (65.2)	593 (67.9)	298 (69.0)	277 (64.7)
Spanish	125 (28.4)	234 (26.8)	107 (24.8)	122 (28.5)
Other	28 (6.4)	46 (5.3)	27 (6.3)	29 (6.8)

^a^ Race and ethnicity were self-reported by the head of household when applying for health insurance coverage.

^b^ The other/unknown race and ethnicity row corresponds to households who opted not to provide a specific race or ethnicity when applying, as well as a small share of households who self-identified as American Indian/Alaska Native or Native Hawaiian or Pacific Islander.

Randomization was performed by the fifth study author (I.M.) using SQL Server Management Studio (Microsoft). In November 2017, we used the Covered California administrative database to create an indicator for whether a household enrolled in marketplace coverage on or before the end of their 60-day special enrollment period window. Race and ethnicity were self-reported by the head of household when applying for health insurance coverage.

To estimate the effect of the intervention, we used linear regression with robust standard errors to account for heteroskedasticity. Statistical significance was defined as a 2-sided *P* < .05. Data were analyzed in November 2017 using Stata SE 15 (StataCorp LLC). We originally undertook this project to inform agency operations; thus, we did not publicly preregister the study, nor did we publicly post a preanalysis plan.

## Results

All 2173 households were included in the analysis with a mean income of 183.4% of the federal poverty level, and 67.0% had a spoken language preference of English. The mean (SD) age of the head of household was 32.9 (15.2) years. Of the 2173 households, 1280 (58.9%) had a female head of household. By the end of the intervention period, 14.5% of the control group enrolled in Covered California (Figure); this low overall take-up rate is consistent with prior marketplace studies.^2,3^ Assignment to the email group did not increase the enrollment rate (−0.5 percentage points; *P* = .82). Assignment to the phone-only group caused a 4.4 percentage point increase (30%) in the enrollment rate (*P* = .08). Assignment to the phone-plus-email group caused a statistically significant 6.9 percentage point increase (47%) (*P* = .008). The difference between the phone and phone-plus-email group was not statistically significant.

**Figure.**
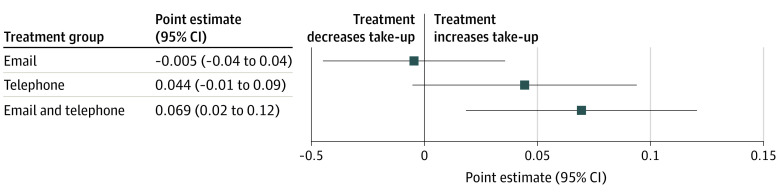
Effect of Treatment Assignment on Covered California Enrollment Rate

## Discussion

As states weigh different approaches to prepare for the end of the COVID-19 public health emergency, this randomized clinical trial found that email reminders had no effect on enrollment, though personalized phone calls paired with email reminders significantly increased ACA enrollment among households losing Medicaid, suggesting that more resource-intensive strategies will be needed to facilitate coverage transitions. Study limitations included small sample sizes, which limit this study’s ability to estimate subgroup effects with precision.

## Notes

Supplement 1.
Trial Protocol.

Supplement 2. **eFigure.** CONSORT flow diagram

Supplement 3.
Data Sharing Statement.
