# Supplementary material for: Effect of Personalized Outreach on Medicaid to Marketplace Coverage Transitions: A Randomized Clinical Trial
Source: JAMA Health Forum. 2022 Oct 14;3(10):e223616. doi: 10.1001/jamahealthforum.2022.3616 (PMC9568803; doi:10.1001/jamahealthforum.2022.3616)
Supplement: Supplement 1. — Trial Protocol. [file jamahealthforum-e223616-s001.pdf]

CENTER FOR DATA INSIGHTS AND INNOVATION
CALIFORNIA HEALTH & HUMAN SERVICES AGENCY

View xForm - Determination for Exempt Research / Not Research

Use this form to request a determination for exempt research or not research

Data entry - Submitted 04/02/2022 10:20 PM ET by Andrew Feher, PhD

Study Personnel

Submitter

Andrew Feher, PhD
Email: andrew.feher@covered.ca.gov **Business:** 818-429-8860

Please complete the questions below. If while trying to complete those questions, personnel are not found by their email address, you can add them in the system by completing the 'new contact form'. Click on the form and complete it. You should then be able to add them in the subsequent questions.

User had the option to start a different form here.

Enter the Principal Investigator's email address

Andrew Feher, PhD
Email: andrew.feher@covered.ca.gov **Business:** 818-429-8860

Choose the PI's institutional affiliation

Other

Click on add contact, and enter the email address for the co-principal investigator for this project. If there are multiple co-principal investigators repeat this action for all co-PIs. If there are no co-PIs for this project, skip this question.

No answer provided.

Enter the administrative contact's email address. If you are the administrative contact, enter your email address.

Andrew Feher, PhD
Email: andrew.feher@covered.ca.gov **Business:** 818-429-8860

Enter the email address of the responsible official for this project. The RO cannot be the same person as the principal investigator (PI). The RO must be one to two levels above the PI.

Mike McKinney

Email: mike.mckinney@covered.ca.gov **Business:** (916) 228-8425

Click on add contact, and enter the email address for the any other research staff that should receive notifications about this project. Repeat this action for all other research staff not previously provided on this screen that should receive notifications about this project. If you have no additional research staff, you may skip this question.

Isaac Menashe, MPP

Email: Isaac.Menashe@covered.ca.gov **Business:** (916) 228-8357

Study Information

A request may be made for CPHS to determine if the project is considered research. Also, a request may be made for CPHS to decide if a research project may be exempt from CPHS review. Both determinations may be requested at the same time. If either determination is made, the project is not required to be approved or renewed by CPHS. After the initial determination, if the project does change to a point that it may be research or not be exempt research, another request must be submitted to CPHS for a determination prior to implementing the changes. If your institution is classified as a for-profit, CPHS may not be able to review your project unless you are able to affiliate with a qualifying organization. Complete this form to determine if your project qualifies for exempt review or to be determine not human subjects research.

Indicate the determination you are requesting

Exempt Research Determination

Select which of the vulnerable populations will be involved in this research. If no vulnerable populations will be involved, choose 'not applicable'.

Note that minors is defined in the United States as under 18 years of age. If research is conducted outside the United States, a minor is under the age of majority in the country(ies) where research is to be conducted. Consult local law for further information about the age of majority in that country.

Note that a “Prisoner" is defined by Department of Health and Human Services regulations , 45 Code of Federal Regulations (CFR) part 46.303(c), as "any individual involuntarily confined or detained in a penal institution. The term is intended to encompass individuals sentenced to such an institution under a criminal or civil statute, individuals detained in other facilities by virtue of statutes or commitment procedures which provide alternatives to criminal prosecution or incarceration in a penal institution, and individuals detained pending arraignment, trial, or sentencing." Patients residing in state mental hospitals in lieu of being imprisoned are considered prisoners. Parolees who are legally required to stay in a transition facility, such as a half-way house or drug-treatment facility, are also considered prisoners.

Not applicable

Indicate the primary site at which the research will be conducted.

Other

Indicate the type of institution you are affiliated with.

Governmental

Please enter the project title

Using Emails and Personalized Phone Calls to Increase Affordable Care Act Marketplace Enrollment Among Households Losing Medicaid

Indicate the study procedures involved in this research. Check all that apply

Program Evaluations

Study Details

Provide the rationale for why this request is being submitted to CPHS for approval (e.g., involvement of state data, state funding or state research staff).

This randomized evaluation will examine existing information from consumers in the Covered California administrative database from 2017. The project involves state research staff as well as state administrative data.

Provide a brief, non-technical description of the project, including a summary of the purpose and goals, project design and procedures.

Since its passage in 2010, the Affordable Care Act (ACA) has helped reduce the uninsured rate to record lows, but changes in insurance coverage over time, known as churn, remain a concern. A recent survey found that nearly 25 percent of respondents reported a change in coverage over the previous twelve months. Among the most common reasons for churn is the loss of Medicaid eligibility, placing low-income populations at risk of a coverage gap. To date, little evidence exists on effective strategies states can use to facilitate Medicaid to Marketplace coverage transitions, an issue that has become more pressing amid projections that upwards of 15 million people could lose Medicaid eligibility once the COVID-19 public health emergency expires.

To address this gap and to inform Marketplace administrators, during a Special Enrollment Period (SEP) in 2017 in California's ACA Marketplace, we conducted a randomized controlled trial to examine the effect of email reminders, personalized telephone outreach, as well as the combination of the two forms of outreach on ACA enrollment among households who recently lost Medicaid and became eligible for subsidized Marketplace coverage.

During the SEP at the end of August 2017, we randomly assigned households to one of four arms based on the last digit of their household identifier: a control group assigned to receive no outreach beyond an initial eligibility determination notice; an email-only group assigned to receive an initial eligibility determination plus email reminders about signing up for marketplace coverage; a phone-only group assigned to receive an initial eligibility determination plus a phone call offering enrollment assistance from a service center representative (SCR); a phone + email group assigned to receive an initial eligibility determination, email reminders about signing up for marketplace coverage and a phone call offering enrollment assistance.

We subsequently obtained administrative enrollment data from Covered California to create an indicator for whether a household enrolled in Marketplace coverage on or before the end of their 60-day SEP enrollment window. We will use regression models to estimate the effect of the different treatments on health insurance enrollment.

Provide a brief description of any data/specimens that will be used and the involvement of human subjects in the study.

For this study, we will use existing Covered California administrative data from the 2017 coverage year for the consumers who are part of the intervention; the data include demographic details, eligibility details and enrollment information.

If you wish to attach a list of variables, do so here. If you listed the variables above, skip this question.
No answer provided.

Describe the data elements/specimens to be used or collected and the source(s) of data/specimens. List the variables to be used in this project.

For this study, we will use existing Covered California administrative data from the 2017 coverage year for the consumers who are part of the intervention; the data include demographic details, eligibility details and enrollment information.

Does your study require recruitment (materials/data collection tools)?

No

Indicate the number of subjects or their data that will be involved in the study and the geographic areas to be covered.

The study will use administrative data for approximately 2,200 households who lost eligibility for Medicaid and became eligible for subsidized coverage through Covered California.

Provide a brief description of the end product, such as a report or article in a peer-reviewed journal, and the plan for disseminating the findings.

The end-product will be a summary of key findings presented to Covered California policymakers and a manuscript prepared for submission to a peer-reviewed academic journal.

Does the researcher collect or obtain the data or specimens that are considered personal information? CPHS defines personal information as being any of the following 18 Health Information Portability and Accountability Act identifiers.

Yes

Check all of the identifiers that apply to your project
Names
Telephone numbers
Any elements of dates (other than year) for dates directly related to an individual, including birth date, admission date, discharge date, date of death. For ages over 89: all elements of dates (including year) indicative of such age, except that such ages and elements may be aggregated into a single category of age 90 and older
Geographic subdivisions smaller than a state (except the first three digits of a zip code if the geographic unit formed by combining all zip codes with the same three initial digits contains more than 20,000 people and the initial three digits of a zip code for all such geographic units containing 20,000 or fewer people is changed to 000).
Electronic mail addresses

Determination of Exempt Questions

Does this project involve any of the following? If so, check all that apply or choose 'none of the above'.

None of the above

Categories of exempt research activities

(1) Research conducted in established or commonly accepted educational settings, involving normal educational practices, such as research on regular and special education instructional strategies or research on effectiveness of or comparison among instructional techniques, curricula or classroom management methods.

(2) Research involving only the use of educational tests, survey procedures, interview procedures or observation of public behavior unless:

> **a) Information is recorded in such a manner that subjects can be identified directly or through identifiers; and**
> **b) Any disclosure of responses outside the research could reasonably place the subjects at risk of criminal or civil liability or be damaging to the subjects' financial standing, employability, or reputation.**

Note: This exemption is not allowed for research involving surveys or interviews with children (up to 18 years) or observation of public behavior of children if the investigator interacts with the children.

(3) Research involving the use of educational tests (cognitive, diagnostic, aptitude, achievement), survey procedures, interview procedures, or observation of public behavior if:

> **(i) Human subjects are elected or appointed officials or candidates for public office or**
>
> **(ii) Federal laws require, without exception, that the confidentiality of personally identifiable information will be maintained throughout the research and thereafter**

(4) Research involving the collection or study of existing data, documents, records, pathological or diagnostic specimens. If:

> **(i) The sources are publicly available**
>
> **(ii) The information is recorded by the investigator in such a manner that subjects cannot be identified, directly or through identifiers linked to the subject.**

(5) Research and demonstration projects which are conducted by or subject to the approval of a federal department or agency head, and are designed to study, evaluate or otherwise examine: (Please note that projects that are federally funded but not directly supervised by a federal department or agency head do not qualify for this exemption)

> **(i) Public benefit or service programs**
>
> **(ii) Procedures for obtaining benefits or services under those public benefit or service programs**
>
> **(iii) Possible changes in or alternatives to public benefit or service programs or**
>
> **(iv) Possible changes in methods or levels of payment for benefits**

or services under public benefit or service programs

(6) Taste and food quality evaluation and consumer acceptance studies. If:

(i) Wholesome foods without additives are consumed or

(ii) The food consumed contains a food ingredient at or below the level and for a use found to be safe, or agricultural chemical or environmental contaminant at or below levels found to be safe by FDA or approved by the EPA or Food Safety and Inspection Service of USDA?

After reviewing the regulatory definition above for the six exempt categories, indicate which category you are asking for exemption under.

Category 4
Category 5

Provide an explanation for why this project qualifies for this category by addressing the relevant requirements

Category 4: This study qualifies as an exempt research activity because the data we will use is existing Covered California administrative data.

Category 5: This research project is being conducted at the agency's request to help understand strategies for reducing health insurance coverage gaps.

Additional Documents

If you have any additional documents to provide, upload them here.

No answer provided.

To sign this form, enter your IRBManager password. By signing this form, you are indicating that the information within this application is accurate and reflects the proposed research.

Signed Saturday, April 2, 2022 10:20:40 PM ET by Andrew Feher, PhD

In order to submit this form, click "Next" and "Submit." At that time, the application will be routed to the Responsible Official (if this is the first submission) for signature.

Copyright ©2000-2022 Tech Software. All Rights Reserved.
/2022.5.6618.0/Release/0b3af4f | GCWAWS1 | 2022-06-10 23:52:04Z
Powered By IRBManager
