# Supplementary material for: Effect of Personalized Outreach on Medicaid to Marketplace Coverage Transitions: A Randomized Clinical Trial
Source: JAMA Health Forum. 2022 Oct 14;3(10):e223616. doi: 10.1001/jamahealthforum.2022.3616 (PMC9568803; doi:10.1001/jamahealthforum.2022.3616)
Supplement: Supplement 2. — eFigure. CONSORT flow diagram [file jamahealthforum-e223616-s002.pdf]

Supplemental Online Content

Ravel K, Ahrary J, Avakian K, Feher A, Menashe I. Effect of personalized outreach on Medicaid to marketplace coverage transitions: a randomized clinical trial. *JAMA Health Forum.* 2022;3(10):e223616. doi:10.1001/jamahealthforum.2022.3616

eFigure. CONSORT flow diagram

This supplemental material has been provided by the authors to give readers additional information about their work.

© 2022 Ravel K et al. *JAMA Health Forum.*

CONSORT Flow Diagram

eFigure

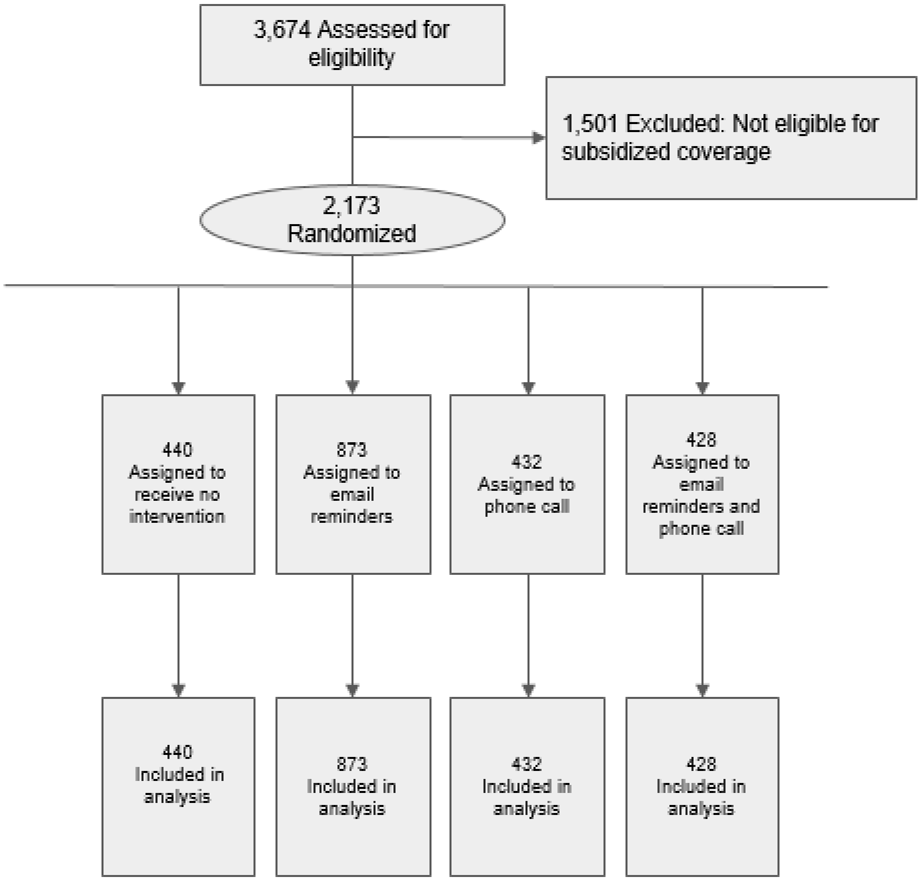

© 2022 Ravel K et al. *JAMA Health Forum.*
