# Supplementary material for: Effect of Personalized Outreach on Medicaid to Marketplace Coverage Transitions: A Randomized Clinical Trial
Source: JAMA Health Forum. 2022 Oct 14;3(10):e223616. doi: 10.1001/jamahealthforum.2022.3616 (PMC9568803; doi:10.1001/jamahealthforum.2022.3616)
Supplement: Supplement 3. — Data Sharing Statement. [file jamahealthforum-e223616-s003.pdf]

Data Sharing Statement

Ravel. Effect of Personalized Outreach on Medicaid to Marketplace Coverage Transitions. *JAMA Health Forum*. Published October 14, 2022. doi:10.1001/jamahealthforum.2022.3616

Data
Data available: Yes
Data types: Deidentified participant data
How to access data: https://osf.io/gpkrc/
When available: beginning date: 08-13-2022

Supporting Documents
Document types: None

Additional Information
Who can access the data: Anyone
Types of analyses: For any purpose
Mechanisms of data availability: Available via OSF: https://osf.io/gpkrc/
